# Ground state cooling of an ultracoherent electromechanical system

**DOI:** 10.1038/s41467-022-29115-9

**Published:** 2022-03-21

**Authors:** Yannick Seis, Thibault Capelle, Eric Langman, Sampo Saarinen, Eric Planz, Albert Schliesser

**Affiliations:** 1 Niels Bohr Institute, University of Copenhagen, Blegdamsvej 17, 2100 Copenhagen, Denmark; 2 Center for Hybrid Quantum Networks (Hy-Q), Niels Bohr Institute, University of Copenhagen, Copenhagen, Denmark

**Keywords:** Optomechanics, Quantum mechanics, Quantum metrology

## Abstract

Cavity electromechanics relies on parametric coupling between microwave and mechanical modes to manipulate the mechanical quantum state, and provide a coherent interface between different parts of hybrid quantum systems. High coherence of the mechanical mode is of key importance in such applications, in order to protect the quantum states it hosts from thermal decoherence. Here, we introduce an electromechanical system based around a soft-clamped mechanical resonator with an extremely high Q-factor (>10^9^) held at very low (30 mK) temperatures. This ultracoherent mechanical resonator is capacitively coupled to a microwave mode, strong enough to enable ground-state-cooling of the mechanics (${\bar{n}}_{\min }=0.76\pm 0.16$). This paves the way towards exploiting the extremely long coherence times (*t*_coh_ > 100 ms) offered by such systems for quantum information processing and state conversion.

## Abstract (web summary)

’Systems with long coherence times are extremely important for the processing of quantum information. To this end the authors present a system able to cool down a resonator to its quantum mechanical ground state harnessing the large coupling between an ultra-coherent mechanical resonator and a superconducting circuit.’

## Introduction

The field of cavity electromechanics^1,2^ investigates mechanical resonators which are parametrically coupled to radio-frequency or microwave circuits. Analogous to cavity optomechanics^3^, this coupling is at the heart of a broad set of phenomena and techniques of interest in quantum science and technology. They range from ground-state cooling of the mechanics^4–6^, via entanglement and squeezing^7–10^, to coherent microwave-optical^11,12^ (see also ref. ^13^ and references therein) and superconducting qubit-mechanical interfaces^14–17^.

For most of these applications, a long coherence time

1 $${t}_{{{{{{{{\rm{coh}}}}}}}}}=\frac{\hslash Q}{{k}_{{{{{{{{\rm{B}}}}}}}}}{T}_{{{{{{{{\rm{bath}}}}}}}}}}=\frac{1}{{\bar{n}}_{{{{{{{{\rm{th}}}}}}}}}{{{\Gamma }}}_{{{{{{{{\rm{m}}}}}}}}}},$$

of the mechanical system is favorable. Here, *Q* = Ω_m_/Γ_m_ is the mechanical quality factor defined as the ratio of the mechanical (angular) frequency Ω_m_ and its energy decay rate Γ_m_; *T*_bath_ the resonator’s bath temperature; *ℏ* and *k*_B_ the reduced Planck and the Boltzmann constants, respectively; and ${\bar{n}}_{{{{{{{{\rm{th}}}}}}}}}\approx {k}_{{{{{{{{\rm{B}}}}}}}}}{T}_{{{{{{{{\rm{bath}}}}}}}}}/\hslash {{{\Omega }}}_{{{{{{{{\rm{m}}}}}}}}}$ is the equivalent occupation of the thermal bath.

For state-of-the-art electromechanical systems operated at millikelvin temperatures, typical Q-factors are ≲10^7^ and coherence times are at most 1 ms. This applies to a wide variety of systems, including aluminum vacuum gap capacitors^7–10,18,19^, metallized silicon nitride membranes^6,11,20,21^ and strings^22^, quantum acoustic devices^14,16^, as well as piezoelectrically coupled nanophononic crystals^15,17^. As a notable exception, *Q* ≈ 10^8^ has been reported for a metallized silicon nitride membrane in 2015^23^. Its enhanced performance over similar devices^6,11,20,21^ might be linked to its particularly low operation temperature (~10 mK) and frequency (~100 kHz)—which, among other things, can make operation in the simultaneously overcoupled and resolved-sideband regime challenging.

On the other hand, recent progress in the design of mechanical systems has allowed reaching quality factors in excess of 10^9^ at mega- to gigahertz frequencies^24–29^. At millikelvin temperatures, such ultracoherent mechanical devices can reach *t*_coh_ > 100 ms, some two orders of magnitude beyond the typical performance of state-of-the-art devices (provided excess dephasing^27^ is not an issue). However, so far, the mechanics’ coupling to microwave modes has either been extremely weak^24^, or absent because of lacking functionalization through e.g., metallization^25–29^. For this reason, these mechanical systems could not yet be harnessed in electromechanics.

Here, we realize an ultracoherent electromechanical system based on a soft-clamped silicon nitride membrane^25^. Following earlier work^5,6,11,23^, we functionalize it with a superconducting metal pad. This allows coupling it to a microwave resonator to implement the standard opto-mechanical Hamiltonian

2 $${\hat{H}}_{{{{{{{{\rm{int}}}}}}}}}=\hslash {g}_{0}{\hat{a}}^{{{{\dagger}}} }\hat{a}\big(\hat{b}+{\hat{b}}^{{{{\dagger}}} }\big),$$

as shown in previous works^1^. Here, *g*_0_/2*π* is the microwave frequency shift due to the zero-point fluctuation of the mechanical resonator, $\hat{a}$($\hat{b}$) is the photon (phonon) annihilation operator. Under a strong pump, the system is populated by a mean coherent field around which the Hamiltonian can be linearized to

3 $${\hat{H}}_{{{{{{{{\rm{int}}}}}}}}}\approx \hslash {g}_{0}\sqrt{n}\left(\delta {\hat{a}}^{{{{\dagger}}} }+\delta \hat{a}\right)\big(\delta \hat{b}+\delta {\hat{b}}^{{{{\dagger}}} }\big),$$

where *n* is the mean photon number in the cavity, and the annihilation operators are here small displacements around a mean coherent field. In this case, well-established concepts and methods of optomechanics as described, e.g., in ref. ^3^ apply. In our work, we realize sufficient coupling strength to cool the mechanical mode to its quantum mechanical ground state. This implies that we have achieved a quantum cooperativity *C*_q_ > 1 (ref. ^3^) and heralds the possibility to deploy soft-clamped mechanical resonators for applications in quantum electromechanics.

## Results

### Electromechanical system

**Fig. 1:**
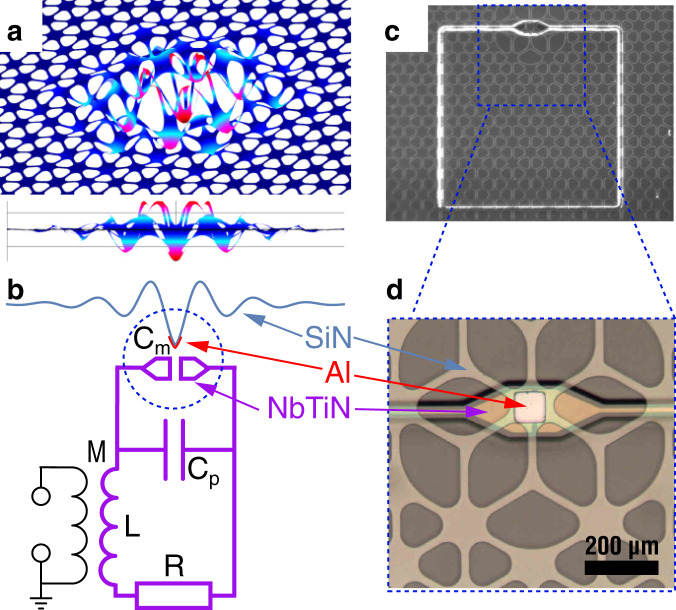
Electromechanical system. **a** Bird’s (top) and side (bottom) view of the simulated displacement of the mechanical mode localized at the defect in the phononic crystal patterned into a silicon nitride (SiN) membrane. False-color indicates displacement amplitude from small (blue) to large (red). **b** The membrane defect is metallized with a pad of aluminum (Al) and brought into proximity of two electrode pads on a different chip, thereby forming a mechanically compliant capacitance *C*_m_. This capacitor is part of a microwave `loop-gap' resonator made from the superconductor NbTiN, together with a parallel parasitic capacitance *C*_p_, inductivity *L* and resistance *R*. Microwave power is coupled into this circuit through the mutual inductance *M*. **c** Gray-scale optical micrograph (top view) of the flip-chip, in which the microwave loop-gap resonator (bright square) shines through the largely transparent patterned membrane. **d** Color zoom onto the mechanically compliant capacitor, showing the square Al metallization on the patterned membrane above the NbTiN capacitor pads.

The system studied here is shown in Fig. 1. It consists of a 63-nm thick soft-clamped membrane made of silicon nitride^25^. A square portion of its central defect (an area of ~60 × 60 μm^2^) is covered with a 50-nm thick layer of aluminum. This superconducting pad is placed, using a flip-chip assembly, closely above the capacitive electrodes of a planar loop-gap resonator fabricated from a 100-nm thick layer of NbTiN, forming a resonant LC circuit. The motion of the metallized membrane modulates the capacitance and in turn the resonance frequency of the microwave circuit, thereby forming a canonical electromechanical system^5,6,11^.

The device is read out by inductive coupling to a coaxial transmission line and is placed on a mechanical damper, for vibration isolation^30^, mounted on the mixing chamber plate of a dilution refrigerator (see “Methods” for details). From microwave reflection measurements performed by the vector network analyzer, we extract a cavity resonance frequency *ω*_c_/2*π* = 8.349 GHz, a total linewidth *κ*/2*π* = 240 kHz and an outcoupling efficiency *η* = *κ*_ex_/*κ* ~ 0.8. With a mechanical mode at Ω_m_/2*π* = 1.486 MHz, the system is well sideband-resolved (*κ* ≪ Ω_m_).

**Fig. 2:**
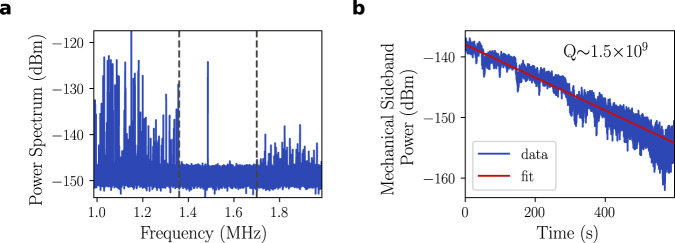
Mechanical properties. **a** Thermal noise spectrum showing a large bandgap (whose limits are indicated with dashed lines) around the mode of interest at ~1.5 MHz. **b** Mechanical ringdown of the defect mode of a metallized membrane at cryogenic temperature, yielding an ultrahigh quality factor.

The soft-clamped membranes utilized in this work represent a new design of phononic membrane resonators, one which we find to have superior characteristics for electromechanical functionalization. Each membrane of this new design is referred to as a ‘Lotus,’ inspired by the resemblance of the defect-defining perforations to the large petals of various species of lotus flowers. Not only do we observe that Lotus-class designs possess larger bandgaps, but they are capable of localizing a single out-of-plane mechanical mode centered in that enlarged bandgap, with maximum amplitude at the center of the defect. Importantly, this single mode remains well-isolated from the bandgap edges after aluminum metallization, as shown in Fig. 2. Finally, we find such metallized lotuses to be able to yield ultrahigh mechanical quality factors in excess of 10^9^ at cryogenic temperature, as measured by energy ringdown (see Fig. 2).

### Calibrations

**Fig. 3:**
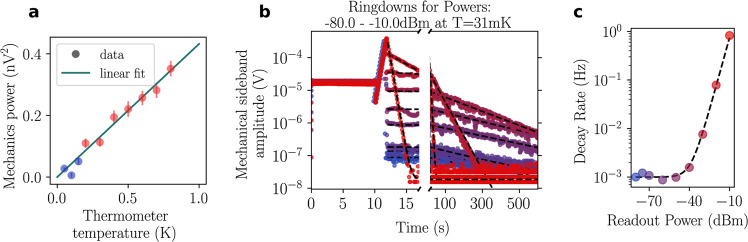
Electromechanical calibration. **a** The mechanical occupation is calibrated by thermal anchoring at temperatures above 200 mK. The linear relationship between the area of the mechanical peak in the spectrum and the sample holder temperature confirms that the mechanics is thermalized. Only the red data are used for the fit (see main text). Error bars are std. dev. of the mechanical sideband area fits. **b** A mechanical energy ringdown series measured as function of the applied cooling power, measured at 30 mK. Overlayed temporal series show repeatable initialization of the mechanical energy (up to ~12 s) and increasing decay rates as the cooling power is turned up. **c** The fit of mechanical decay rates gives the intrinsic decay rate Γ_m_, without dynamical backaction, and the corner power *P*_0_, where the cooling rate Γ_e_(*P*_0_) is equal to Γ_m_. Points' color code is the same as in panel (**b**).

We establish the phonon occupation of the mechanical resonator via its equilibration to the controlled thermal bath provided in the cryostat. We drive the electromechanical system with a tone at angular frequency *ω*_p_, red-detuned from the cavity resonance Δ ≡ *ω*_p_ − *ω*_c_ ≈ − Ω_m_ < 0 at a fixed, low power (−45 dBm at the source) such that dynamical backaction^3^ is negligible. Then, after further amplification and carrier cancellation (see “Methods”), we measure the spectral area occupied by the mechanical sideband (i.e., the total microwave power) around the angular frequency *ω*_p_ + Ω_m_ for a range of sample temperatures as measured by the cryostat thermometer. At temperatures above ~200 mK (see Fig. 3a), we observe a linear relationship between temperature and mechanical sideband area. This proportionality is interpreted as the sample being in a thermal equilibrium with the mixing chamber plate. Using Bose-Einstein statistics for thermal states ${\bar{n}}_{{{{{{{{\rm{th}}}}}}}}}={({e}^{\hslash {{{\Omega }}}_{{{{{{{{\rm{m}}}}}}}}}/{k}_{{{{{{{{\rm{B}}}}}}}}}{T}_{{{{{{{{\rm{bath}}}}}}}}}}-1)}^{-1}$, we extract a calibration constant between mechanical sideband area and mechanical occupation in quanta. At temperatures below ~200 mK, dynamical backaction is small but nonnegligible (≲15%). This has been corrected for, together with the temperature-dependent microwave and mechanical damping (see Supplementary Material). Figure 3a shows the resulting thermalization of the mechanical oscillator to the base plate of the cryostat. From this analysis, we infer that at the lowest cryostat temperature of 30 mK, the mechanical mode is coupled to a bath at *T*_bath_ ≈ 80 mK.

Next, we calibrate the dynamical backaction by performing mechanical ringdown measurements under red-detuned microwave drives with varying powers. Figure 3b shows superimposed ringdown sequences under increasing microwave power. For these measurements, we initialize the mechanics into a large coherent state by phase modulation of the red-detuned pump (duration 10 s), then amplify this coherent state by placing the pump on the blue side of the cavity (duration 1.75 s). Finally, we let the mechanics ringdown for 600 s with the red-detuned microwave pump at power *P* varying from −80 to −10 dBm (at the output of the signal generator). We fit the ringdowns to exponential decays where the time constants are the inverse angular decay rates ${{{\Gamma }}}_{{{{{{{{\rm{eff}}}}}}}}}^{-1}$.

The resulting decay rates as function of pump power are shown in Fig. 3c, together with a fit using the model

4 $${{{\Gamma }}}_{{{{{{{{\rm{eff}}}}}}}}}(P)={{{\Gamma }}}_{{{{{{{{\rm{m}}}}}}}}}+{{{\Gamma }}}_{{{{{{{{\rm{e}}}}}}}}}(P)={{{\Gamma }}}_{{{{{{{{\rm{m}}}}}}}}}\left(1+\frac{P}{{P}_{{{{{{{{\rm{0}}}}}}}}}}\right).$$

Here, Γ_m_ is the intrinsic loss rate of the mechanical resonator, while Γ_e_(*P*) is the damping imparted by the dynamical backaction of the microwave mode^3^. We introduce *P*_0_ as the power at which the pump-induced decay is equal to the intrinsic decay rate Γ_m_. Note that *P*_0_ depends on the cavity lineshape and the pump detuning from cavity resonance. At a cryostat temperature of 30 mK, we extract Γ_m_/2*π* = 1.0 mHz, a quality factor *Q*_m_ = Ω_m_/Γ_m_ = 1.5 × 10^9^ and *P*_0_ = −38.7 dBm from the dataset shown in Fig. 3. We note that the quality factor is dependent on the sample temperature (see Supplementary for a systematic analysis). We perform ground-state cooling at the same temperature, allowing us to use the Γ_m_ and Γ_e_(*P*) obtained from this fit as fixed parameters in all further analysis.

Finally, from a standard calibration technique detailed in ref. ^31^, we extract a single-photon coupling rate *g*_0_/2*π* = 0.89 ± 0.11 Hz. From the same calibration, we obtain an overall electronic gain between the sample and the spectrum analyzer. From a complementary measurement of transmission of the entire setup, we can infer an attenuation of (66.5 ± 1) dB between the signal source and the sample. This means that for the highest source power of 10 dBm, the power at the device input is −56.5 dBm and the cavity is populated with 3.3 × 10^7^ microwave photons.

### Ground-state cooling

To reduce the mechanical occupation of our mechanical resonator, we place a strong coherent pump on the red sideband of the microwave cavity (Δ = −Ω_m_), in the same experimental conditions with which we calibrated the phonon occupation (see the “Calibrations” section).

In the resolved-sideband limit (*κ* ≪ Ω_m_), which we reach in this experiment, and close to the cavity frequency (∣*ω* − *ω*_c_∣ ≪ *κ*), the microwave power spectral density in units of noise quanta is then:

5 $$S[\omega ]={n}_{{{{{{{{\rm{add}}}}}}}}}+4\eta \left(\tilde{n}+1/2\right)+\eta {{{\Gamma }}}_{{{{{{{{\rm{m}}}}}}}}}{{{\Gamma }}}_{{{{{{{{\rm{e}}}}}}}}}\frac{{\bar{n}}_{{{{{{{{\rm{th}}}}}}}}}+\frac{1}{2}-\left(2+\frac{{{{\Gamma }}}_{{{{{{{{\rm{e}}}}}}}}}}{{{{\Gamma }}}_{{{{{{{{\rm{m}}}}}}}}}}\right)\left(\tilde{n}+\frac{1}{2}\right)}{{\left({{{\Gamma }}}_{{{{{{{{\rm{m}}}}}}}}}+{{{\Gamma }}}_{{{{{{{{\rm{e}}}}}}}}}\right)}^{2}/4+{\big(\omega -{\omega }_{{{{{{{{\rm{p}}}}}}}}}-{{{\Omega }}}_{{{{{{{{\rm{eff}}}}}}}}}\big)}^{2}},$$

where we have defined $\tilde{n}=\eta {n}_{c}+\left(1-\eta \right){n}_{0}$, with *η* = *κ*_c_/*κ*, *κ*_c_ the coupling rate to the microwave cavity, *n*_c_ the microwave noise occupation coming from either the pump phase noise or the cavity frequency noise, *n*_0_ the noise occupation of the microwave thermal environment (which is negligible in the considered experimental conditions). In the above expression, Ω_eff_/2*π* is the effective mechanical frequency including the frequency shift induced by the dynamical backaction. The measured microwave spectrum is composed of three parts: the background noise *n*_add_, which is due to the HEMT amplifier, the microwave noise coming from the cavity, which is a Lorentzian whose width is the microwave loss rate *κ*, and the mechanical noise transduced into microwave noise *via* the electromechanical coupling. This mechanical feature is a Lorentzian of width Γ_eff_ = Γ_m_ + Γ_e_.

At low cooperativity *C* ≈ Γ_e_/Γ_m_ ≪ 1, the signal, divided by the electromechanical gain *η*Γ_e_/Γ_m_ is simply a Lorentzian whose area is proportional to the mechanical bath occupation ${\bar{n}}_{{{{{{{{\rm{th}}}}}}}}}\gg \tilde{n}$. This is the regime where we performed the calibrations presented in the section “Calibrations”.

At a higher cooperativity *C* ≈ Γ_e_/Γ_m_ ≫ 1, the rate at which phonons are extracted from the resonators initially exceeds the rate at which new phonons are entering the resonator *via* the mechanical thermal bath. A new equilibrium is established at a reduced temperature of the mechanical resonator, corresponding to a reduced effective occupation $\bar{n} \, < \, {\bar{n}}_{{{{{{{{\rm{th}}}}}}}}}$. This appears as a decrease of the area under the mechanical spectrum.

**Fig. 4:**
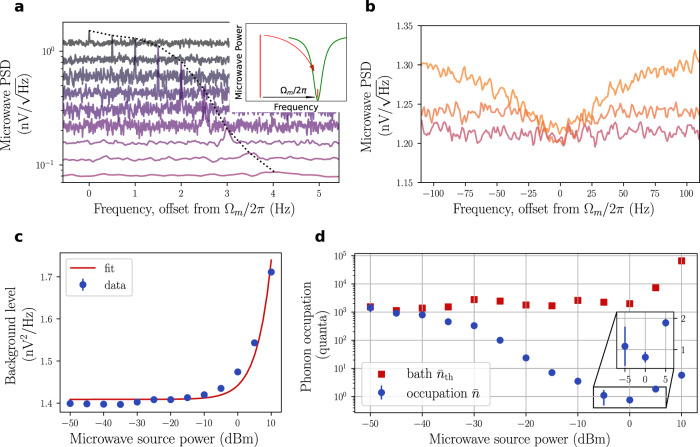
Sideband cooling of the mechanics to its motional ground state. **a** Mechanical power spectral density (PSD) around the defect mode as the cooling power is increased: the peak first increases in height with measurement gain, then visibly broadens as cooling takes place. For readability, spectra are artificially offset downward and to the right in equal steps respectively as the power is increased. The dotted line is a guide to the eye for the location of the mechanical peaks. The inset depicts the scattering process due to the mechanics, which creates a sideband (small vertical red line) in the microwave cavity (green dip) at an angular frequency Ω_*m*_ above the pump tone (tall vertical red line). **b** At high pump powers, the cavity is populated due to the microwave phase noise: the noise occupancy is visible in the ‘squashing’ of the mechanical feature. **c** The increased background level of the mechanical spectrum allows to extract the cavity occupation $\tilde{n}$. **d** The mechanical occupation, calibrated in number of motional quanta, reaches below one phonon by dynamical backaction cooling before being heated up by cavity occupation due to input phase noise. Error bars are std. dev. of the mechanical sideband area fits.

At the highest cooperativities, the microwave noise starts to play a significant role. It originates either from the phase noise of the microwave source or from a cavity frequency noise. We see from Eq. (5) that the observed signal can then be a *negative* Lorentzian. This does not mean that the temperature of the mechanical mode is negative, but rather that the cross spectrum between the microwave noise in the cavity and the microwave noise transduced to mechanical noise changes the shape of the resulting signal^19^. In this case, inference of the mechanical mode temperature requires the knowledge of the phase noise, which is given by the background of the signal (for Γ_m_ + Γ_e_ ≪ ∣*ω* − *ω*_c_∣ ≪ *κ*)^32^:

6 $$S{[\omega ]}_{{{{\Gamma }}}_{{{{{{{{\rm{m}}}}}}}}}+{{{\Gamma }}}_{{{{{{{{\rm{e}}}}}}}}}\ll | \omega -{\omega }_{{{{{{{{\rm{c}}}}}}}}}| \ll \kappa }={{{{{{{\mathcal{A}}}}}}}}+\alpha P,$$

where *P* is the pump power, $\alpha P=4{\eta }^{2}{n}_{c}\approx 4\eta \tilde{n}$, and ${{{{{{{\mathcal{A}}}}}}}}={n}_{{{{{{{{\rm{add}}}}}}}}}+2\eta +4\eta \left(1-\eta \right){n}_{0}\approx {n}_{{{{{{{{\rm{add}}}}}}}}}$. By fitting the model of Eq. (5) to the experimental spectra comprising both the mechanical feature and the background level, we obtain the parameters ${\bar{n}}_{{{{{{{{\rm{th}}}}}}}}}$ and $\tilde{n}$, respectively, at each power level (see Fig. 4). We can then compute the mechanical occupation as^5^:

7 $$\bar{n}=\frac{{{{\Gamma }}}_{{{{{{{{\rm{m}}}}}}}}}}{{{{\Gamma }}}_{{{{{{{{\rm{m}}}}}}}}}+{{{\Gamma }}}_{{{{{{{{\rm{e}}}}}}}}}}{\bar{n}}_{{{{{{{{\rm{th}}}}}}}}}+\frac{{{{\Gamma }}}_{{{{{{{{\rm{e}}}}}}}}}}{{{{\Gamma }}}_{{{{{{{{\rm{m}}}}}}}}}+{{{\Gamma }}}_{{{{{{{{\rm{e}}}}}}}}}}\tilde{n}.$$

The minimum inferred occupation is ${\bar{n}}_{\min }=0.76\pm 0.16$. This final value is limited by the efficiency of the vibration isolation, which increases the mechanical bath temperature above the thermodynamic temperature (see “Methods”), and the microwave phase noise at the input of the system. Although an increase of the mechanical bath temperature can be observed for pump powers ≥0 dBm, the contribution of the phase noise is still dominant. Placing a microwave cavity filter at the output of the signal generator, absorbing pump phase noise around the electromechanical cavity resonance, did not improve the result. This suggests that the phase noise is limited by cavity noise rather than the phase noise of the microwave source.

## Discussion

The mechanical occupation calibrated in Fig. 3a along with the measured intrinsic mechanical decay rate furthermore allows us to estimate the mechanics’ quantum coherence time. Following Eq. (1), we extract *t*_coh_ ≈ 140 ms. This is three orders of magnitude larger than for state-of-the-art electromechanical systems^10,14^. However, further work will be needed to fully confirm the coherence of the mechanical system, ruling out e.g., excess decoherence by dephasing^27^.

At the highest input powers (*P* = 10 dBm), we achieve a cooperativity $C={{{{{{{\mathcal{O}}}}}}}}(1{0}^{5})$ and an electromechanical damping on the order of Γ_e_/2*π* ~ 80 Hz. However, we estimate that the single-photon coupling rate *g*_0_ might be increased by an order of magnitude by adjustment of the geometry, in particular the gap between the membrane electrode and its counterelectrodes. This would immediately boost the coupling (with ${{{\Gamma }}}_{{{{{{{{\rm{e}}}}}}}}}\propto {g}_{0}^{2}$) and simultaneously alleviate the issues with microwave phase noise. Indeed, *g*_0_/2*π* = 7 Hz and coupling rates well above 100 kHz have been demonstrated in a similar system^6^. The challenge in transferring this result to our system lies in realizing similarly small capacitive gaps in spite of a significantly larger membrane size, posing stringent requirements on wafer flat- and cleanliness.

Potential applications of the platform introduced here include quantum memories for microwave quantum states^18^, where they could replace or supplement less coherent (${t}_{{{{{{{{\rm{coh}}}}}}}}}^{{{{{{{{\rm{MW}}}}}}}}} \sim 10\,{{{{{{{\rm{ms}}}}}}}}$), much more bulky microwave resonators^33^. By combining this with an *opto*-mechanical interface^26^, e.g., by introducing a second defect in the phononic crystal^34^, such systems could form part of an electro-opto-mechanical transducer^11,12^. One of its key figures of merit, namely the number of added noise quanta, falls proportionally with the coherence time of the mechanics^35^. Furthermore, the high mechanical coherence immediately translates to an outstanding force sensitivity. This allows for the microwave mode to be used as a sensitive transducer for the motion induced by the physical system of interest, which could be anything from spins^36–38^ to dark matter^39^. Nominally, the resonant force noise spectral density of the presented device is ${S}_{FF}^{1/2}={(2m{{{\Gamma }}}_{{{{{{{{\rm{m}}}}}}}}}{k}_{{{{{{{{\rm{B}}}}}}}}}T)}^{1/2}\approx 650\,{{{{{{{\rm{zN}}}}}}}}/{{{{{{{{\rm{Hz}}}}}}}}}^{1/2}$, assuming the mode mass of ~15 ng estimated by COMSOL simulations. Finally, the membranes’ extremely long coherence time could enable electromechanical experiments to test fundamental physics. They may, for example, constrain the parameters of collapse models^40^, such as the continuous spontaneous localization model (CSL)^41^, which is based on a nonlinear stochastic extension of the Schrödinger equation. Testing the effects of general relativity on massive quantum superpositions with such systems has also been proposed recently^42^.

## Methods

### Sample fabrication

The planar microwave resonator is a patterned thin film of NbTiN sputter-deposited by Star Cryoelectronics on a high-resistivity silicon wafer from Topsil. The superconductor is patterned with standard UV lithography and etched with an ICP recipe based on SF_6_/O_2_ at low power to avoid resist burning. Aluminum pillars define the flip-chip nominal separation, and we etch a recess into the resonator Si chip using the Bosch process to minimize the risk of the flip-chip contacting anywhere else than at the pillars.

The membrane is made of stoichiometric high-stress silicon nitride patterned with standard UV lithography and etched with a CF_4_/H_2_-based ICP recipe on wafer front and back side. The membrane is released in a hot KOH bath. The membranes are then cleaned in a bath of piranha solution, broken off to individual chips, and metallized by shadow-masked e-beam evaporation of aluminum.

### Reporting summary

Further information on research design is available in the Nature Research Reporting Summary linked to this article.

## Supplementary information

Supplementary Information

Peer review file

Reporting Summary

## Data Availability

The raw data that support the findings of this study are available from the corresponding author upon reasonable request. Processed data representing all the data in the published figures, both from the main text and the Supplementary Material, are available in the Zenodo open repository https://zenodo.org/record/5996595, together with a Jupyter notebook to plot the figures.
