# Supplementary material for: Ground state cooling of an ultracoherent electromechanical system
Source: Nat Commun. 2022 Mar 21;13:1507. doi: 10.1038/s41467-022-29115-9 (PMC8938490; doi:10.1038/s41467-022-29115-9)
Supplement: Supplementary file 1 — Supplementary Information [file 41467_2022_29115_MOESM1_ESM.pdf]

# Supplementary Material for Ground State Cooling of an Ultracoherent Electromechanical System

Yannick Seis[1,2], Thibault Capelle[1,2], Eric Langman[1,2], Sampo Saarinen[1,2], Eric Planz[1,2], and Albert Schliesser[1,2,*]

[1]Niels Bohr Institute, University of Copenhagen, Blegdamsvej 17, 2100 Copenhagen, Denmark
[2]Center for Hybrid Quantum Networks (Hy-Q), Niels Bohr Institute, University of Copenhagen, Copenhagen, Denmark
[*]email: albert.schliesser@nbi.dk

## Contents

**1 Model derivation** **2**
- 1.1 Langevin equations . . . 2
- 1.2 Input noise definition . . . 3
- 1.3 Mechanical state . . . 3
- 1.4 Optical output . . . 5
- 1.5 Spectrum output . . . 5
  - 1.5.1 Shot noise . . . 6
  - 1.5.2 Mechanical noise . . . 6
  - 1.5.3 Cross spectrum . . . 7
- 1.6 Final result . . . 8

**2 System Summary** **8**
- 2.1 Finite element simulations . . . 8

**3 Calibrations** **11**
- 3.1 Example of a cavity fit . . . 11
- 3.2 Noise photons in the cavity . . . 11
- 3.3 Additional mechanical dephasing . . . 12
- 3.4 Gorodetsky calibration . . . 12
- 3.5 Cavity monitoring . . . 14
- 3.6 Mechanical mode damping . . . 16
- 3.7 Cryogenic setup . . . 17
- 3.8 Calibration consistency check . . . 18

# 1 Model derivation

We are going to derive the model following a similar calculus than Weinstein *et al*[1], but in the case of a carrier cancellation, in order to have a model for the squashing observed close to the ground state in a sideband cooling experiment.

## 1.1 Langevin equations

We start from the following Hamiltonian, which is the standard optomechanical Hamiltonian:

$$\hat{H} = \hbar\Omega_m \hat{b}^\dagger \hat{b} + \hbar\omega_c \hat{a}^\dagger \hat{a} + \hbar g_0 \hat{a}^\dagger \hat{a}\left(\hat{b} + \hat{b}^\dagger\right), \tag{S1}$$

where $\omega_c/2\pi$ ($\Omega_m/2\pi$) is the microwave (mechanical) frequency, $g_0/2\pi$ is the microwave frequency shift induced by the zero point motion of the mechanical resonator, and $\hat{a}$ ($\hat{b}$) is the photon (phonon) annihilation operator. The set of Langevin equations deriving from this Hamiltonian are the following:

$$\frac{d}{dt}\hat{a} = \left(-i\omega_c - \kappa/2\right)\hat{a} - ig_0\hat{a}\left(\hat{b} + \hat{b}^\dagger\right) + \sqrt{\kappa_0}\hat{a}_{\mathrm{in,0}} + \sqrt{\kappa_c}\hat{a}_{\mathrm{in,c}} \tag{S2}$$

$$\frac{d}{dt}\hat{b} = \left(-i\Omega_m - \Gamma_m/2\right)\hat{b} - ig_0\hat{a}^\dagger\hat{a} + \sqrt{\Gamma_m}\hat{b}_{\mathrm{in}}, \tag{S3}$$

where $\kappa_0$ ($\kappa_c$) is the intrinsic (coupling) microwave loss rate, $\kappa \underset{\mathrm{def}}{=} \kappa_0 + \kappa_c$ is the total microwave loss rate, $\Gamma_m$ is the mechanical loss rate and $\hat{a}_{\mathrm{in,0}}$, $\hat{a}_{\mathrm{in,c}}$ and $\hat{b}_{\mathrm{in}}$ are the noise bosonic operators associated with those loss channels.

We consider the case of such a system driven by a strong optical pump at the frequency $\omega_p/2\pi$, and consider the following approximations:

$$\hat{a} \approx e^{-i\omega_p t}\left(\alpha + \hat{d}\right) \tag{S4}$$

$$\hat{b} \approx \beta + \hat{c}, \tag{S5}$$

where $\alpha$ ($\beta$) is the amplitude of large coherent field inside the optical (mechanical) oscillator, and $\hat{c}$ ($\hat{d}$) is the annihilation operator corresponding to first order expansions of the field around this mean coherent state.

First, if we define $\hat{a}_r \underset{\mathrm{def}}{=} e^{i\omega_p t}\hat{a}$, we have:

$$\frac{d}{dt}\hat{a}_r = \left(i\Delta - \kappa/2\right)\hat{a}_r - ig_0\hat{a}_r\left(\hat{b} + \hat{b}^\dagger\right) + \sqrt{\kappa_0}\hat{a}_{\mathrm{in,0,r}} + \sqrt{\kappa_c}\hat{a}_{\mathrm{in,c,r}}, \tag{S6}$$

where $\Delta = \omega_p - \omega_c$, $\hat{a}_{\mathrm{in,c,r}} \underset{\mathrm{def}}{=} \hat{a}_{\mathrm{in,c}}e^{i\omega_p t}$, and $\hat{a}_{\mathrm{in,0,r}} \underset{\mathrm{def}}{=} \hat{a}_{\mathrm{in,0}}e^{i\omega_p t}$.

At zeroth order we have:

$$0 = \left(i\Delta - \kappa/2\right)\alpha - ig_0\alpha\left(\beta + \beta^*\right) + \sqrt{\kappa_c}\alpha_{\mathrm{in}} \tag{S7}$$

$$0 = \left(-i\Omega_m - \Gamma_m/2\right)\beta - ig_0|\alpha|^2 \tag{S8}$$

We can show that this system, provided that the incoming intensity is low enough, leads to a solution $(\alpha, \beta)$ that represents the amplitude of the mean field in the cavity.

At first order, we have:

$$\frac{d}{dt}\hat{d} = \left(i\tilde{\Delta} - \kappa/2\right)\hat{d} - ig_0\alpha\left(\hat{c} + \hat{c}^\dagger\right) + \sqrt{\kappa_0}\hat{d}_{\text{in},0} + \sqrt{\kappa_c}\hat{d}_{\text{in,c}} \tag{S9}$$

$$\frac{d}{dt}\hat{c} = \left(-i\Omega_m - \Gamma_m/2\right)\hat{c} - ig_0\left(\alpha\hat{d}^\dagger + \alpha^*\hat{d}\right) + \sqrt{\Gamma_m}\hat{c}_{\text{in}}, \tag{S10}$$

where we have defined $\tilde{\Delta} \underset{\text{def}}{=} \Delta - g_0\left(\beta + \beta^*\right)$. We will now absorb the phase of $\alpha \underset{\text{def}}{=} |\alpha|e^{i\Psi_\alpha}$ in a redefinition of the optical annihilation operator: $\tilde{\hat{d}} \underset{\text{def}}{=} e^{-i\Psi_\alpha}\hat{d}$. We then have:

$$\frac{d}{dt}\hat{d} = \left(i\Delta - \kappa/2\right)\hat{d} - ig\left(\hat{c} + \hat{c}^\dagger\right) + \sqrt{\kappa_0}\hat{d}_{\text{in},0} + \sqrt{\kappa_c}\hat{d}_{\text{in,c}} \tag{S11}$$

$$\frac{d}{dt}\hat{c} = \left(-i\Omega_m - \Gamma_m/2\right)\hat{c} - ig\left(\hat{d}^\dagger + \hat{d}\right) + \sqrt{\Gamma_m}\hat{c}_{\text{in}}, \tag{S12}$$

were the tildes were omitted for clarity, and we defined $g \underset{\text{def}}{=} g_0|\alpha|$.

## 1.2 Input noise definition

The input noise terms are defined as such:

$$\begin{aligned}
\langle\hat{d}_{\text{in},0}(t)\hat{d}^\dagger_{\text{in},0}(t')\rangle &= \left(n_0 + 1\right)\delta(t - t') \\
\langle\hat{d}^\dagger_{\text{in},0}(t)\hat{d}_{\text{in},0}(t')\rangle &= n_0\delta(t - t') \\
\langle\hat{d}_{\text{in,c}}(t)\hat{d}^\dagger_{\text{in,c}}(t')\rangle &= \left(n_c + 1\right)\delta(t - t') \\
\langle\hat{d}^\dagger_{\text{in,c}}(t)\hat{d}_{\text{in,c}}(t')\rangle &= n_c\delta(t - t') \\
\langle\hat{c}^\dagger_{\text{in}}(t)\hat{c}_{\text{in}}(t')\rangle &= \bar{n}_{\text{th}}\delta(t - t') \\
\langle\hat{c}_{\text{in}}(t)\hat{c}^\dagger_{\text{in}}(t')\rangle &= \left(\bar{n}_{\text{th}} + 1\right)\delta(t - t')
\end{aligned} \tag{S13}$$

. Here, $n_c$ will represent the phase noise of the system.

## 1.3 Mechanical state

We place ourselves in the so called *weak coupling regime*, where the power is weak enough to have an optical loss rate well larger than the mechanical loss rate. In this condition, we can compute the mechanical occupation by assuming the optical field in its steady state, which is oscillating at the mechanical frequency. This is done by defining:

$$\hat{d} \approx \hat{d}_+ e^{i\Omega_m t} + \hat{d}_- e^{-i\Omega_m t}, \tag{S14}$$

and that we can assume that $\hat{d}_\pm \approx \hat{d} e^{\mp i\Omega_m t}$. We then have:

$$\frac{d}{dt}\hat{d}_+ = \left(i\left(\Delta - \Omega_m\right) - \kappa/2\right)\hat{d}_+ - ig\left(\hat{c} + \hat{c}^\dagger\right)e^{-i\Omega_m t} + \sqrt{\kappa_0}\hat{d}_{\text{in},0,+} + \sqrt{\kappa_c}\hat{d}_{\text{in,c},+} \tag{S15}$$

$$\frac{d}{dt}\hat{d}_- = \left(i\left(\Delta + \Omega_m\right) - \kappa/2\right)\hat{d}_- - ig\left(\hat{c} + \hat{c}^\dagger\right)e^{i\Omega_m t} + \sqrt{\kappa_0}\hat{d}_{\text{in},0,-} + \sqrt{\kappa_c}\hat{d}_{\text{in,c},-}. \tag{S16}$$

In the steady state, we have $\frac{d}{dt}\hat{d}_\pm \sim 0$. We also have $\left(\hat{c} + \hat{c}^\dagger\right)e^{i\Omega_m t} \sim \hat{c}e^{i\Omega_m t}$ and $\left(\hat{c} + \hat{c}^\dagger\right)e^{-i\Omega_m t} \sim \hat{c}^\dagger e^{-i\Omega_m t}$. This allows to retrieve the values:

$$\hat{d}_+ = \mathcal{A}_-\left[-ig\hat{c}^\dagger + \hat{\mathcal{N}}_{\text{in}}\right]e^{-i\Omega_m t} \tag{S17}$$

$$\hat{d}_- = \mathcal{A}_+\left[-ig\hat{c} + \hat{\mathcal{N}}_{\text{in}}\right]e^{i\Omega_m t}, \tag{S18}$$

where we have defined $\mathcal{A}_\pm \underset{\text{def}}{=} \frac{1}{\kappa/2 - i(\Delta \pm \Omega_m)}$, and $\hat{\mathcal{N}}_{\text{in}} \underset{\text{def}}{=} \sqrt{\kappa_0}\hat{d}_{\text{in},0} + \sqrt{\kappa_c}\hat{d}_{\text{in,c}}$. We can inject this equation in the mechanical Langevin equation. In the limit of a high quality factor, where $\hat{c}$ is oscillating mainly at $\Omega_m$, we have:

$$\frac{d}{dt}\hat{c} = \left(-i\Omega_m - \Gamma_m/2\right)\hat{c} + g^2\left[\mathcal{A}_-^* - \mathcal{A}_+\right]\hat{c} - ig\left[\mathcal{A}_+\hat{\mathcal{N}}_{\text{in}} + \mathcal{A}_-^*\hat{\mathcal{N}}_{\text{in}}^\dagger\right] + \sqrt{\Gamma_m}\hat{c}_{\text{in}}. \tag{S19}$$

We can rewrite the above equation in:

$$\frac{d}{dt}\hat{c} = \left(-i\Omega_{\text{eff}} - \Gamma_{\text{eff}}/2\right)\hat{c} - ig\left[\mathcal{A}_+\hat{\mathcal{N}}_{\text{in}} + \mathcal{A}_-^*\hat{\mathcal{N}}_{\text{in}}^\dagger\right] + \sqrt{\Gamma_m}\hat{c}_{\text{in}}, \tag{S20}$$

where we have:

$$\Omega_{\text{eff}} \underset{\text{def}}{=} \Omega_m + \Omega_e \tag{S21}$$

$$\Gamma_{\text{eff}} \underset{\text{def}}{=} \Gamma_m + \Gamma_e \tag{S22}$$

$$\Omega_e \underset{\text{def}}{=} -g^2\text{Im}\left[\mathcal{A}_-^* - \mathcal{A}_+\right] \tag{S23}$$

$$\Gamma_e \underset{\text{def}}{=} -2g^2\text{Re}\left[\mathcal{A}_-^* - \mathcal{A}_+\right]. \tag{S24}$$

This can be integrated in:

$$\hat{c}(t) = \int_{-\infty}^{t} dt' e^{\left(i\Omega_{\text{eff}} + \Gamma_{\text{eff}}/2\right)\left(t'-t\right)}\left\{-ig\left[\mathcal{A}_+\hat{\mathcal{N}}_{\text{in}} + \mathcal{A}_-^*\hat{\mathcal{N}}_{\text{in}}^\dagger\right] + \sqrt{\Gamma_m}\hat{c}_{\text{in}}\right\}(t') \tag{S25}$$

## 1.4 Optical output

If we define the operator $\hat{X} \underset{\text{def}}{=} \left(\hat{c} + \hat{c}^\dagger\right)$, we have for the optical field:

$$\frac{d}{dt}\hat{d} = \left(i\Delta - \kappa/2\right)\hat{d} - ig\hat{X} + \hat{\mathcal{N}}_{\text{in}}, \tag{S26}$$

which can be integrated in:

$$\hat{d} = \int_{-\infty}^{t} dt' e^{(-i\Delta+\kappa/2)\left(t'-t\right)} \left(-ig\hat{X} + \hat{\mathcal{N}}_{\text{in}}\right)(t'). \tag{S27}$$

Using the input-output relation:

$$\hat{d}_{\text{out}} = -\hat{d}_{\text{c,in}} + \sqrt{\kappa_c}\hat{d}, \tag{S28}$$

which, after carrier cancellation is changed into:

$$\hat{d}_{\text{out}} = +\sqrt{\kappa_c}\hat{d}, \tag{S29}$$

and assuming that we measure the operator $\hat{I} \underset{\text{def}}{=} \hat{d}_{\text{out}} + \hat{d}^\dagger_{\text{out}}$, we have:

$$\hat{I}(t) = +\sqrt{\kappa_c}\int_{-\infty}^{t} dt' e^{\kappa/2\left(t'-t\right)} \Bigg( g\hat{X}(t')\left(-ie^{-i\Delta\left(t'-t\right)} + ie^{i\Delta\left(t'-t\right)}\right) \tag{S30}$$

$$+ e^{-i\Delta\left(t'-t\right)}\hat{\mathcal{N}}_{\text{in}}(t') \tag{S31}$$

$$+ e^{i\Delta\left(t'-t\right)}\hat{\mathcal{N}}^\dagger_{\text{in}}(t')\Bigg) \tag{S32}$$

## 1.5 Spectrum output

We will use the same spectrum definition as in [1], namely:

$$\mathcal{S}[\omega] \underset{\text{def}}{=} \frac{1}{2}\int_{-\infty}^{\infty} dt \langle I(t)I(0) + I(0)I(t)\rangle e^{-i\omega t} \tag{S33}$$

We first compute the measured power:

$$\frac{1}{2}\langle\{I(t), I(0)\}\rangle = \mathcal{P}_{\text{shot,shot}} + \mathcal{P}_{\text{mech,mech}} + \mathcal{P}_{\text{mech,shot}}, \tag{S34}$$

where $\{A, B\} \underset{\text{def}}{=} AB + BA$, $\mathcal{P}_{\text{shot,shot}}$ is the contribution from the optical noise, $\mathcal{P}_{\text{mech,mech}}$ is the contribution from the mechanical noise and $\mathcal{P}_{\text{mech,shot}}$ is the cross spectrum.

### 1.5.1 Shot noise

We have:

$$
\begin{aligned}
\mathcal{P}_{\text{shot,shot}} = \frac{1}{2}\langle\{\sqrt{\kappa_c}\int_{-\infty}^{t} dt' e^{\kappa/2\left(t'-t\right)} \Bigg[ & e^{-i\Delta\left(t'-t\right)}\hat{\mathcal{N}}_{\text{in}}(t') \\
& + e^{i\Delta\left(t'-t\right)}\hat{\mathcal{N}}_{\text{in}}^{\dagger}(t')\Bigg], \\
& + \sqrt{\kappa_c}\int_{-\infty}^{0} dt' e^{\kappa/2t'}\Bigg[e^{-i\Delta t'}\hat{\mathcal{N}}_{\text{in}}(t') \\
& + e^{i\Delta t'}\hat{\mathcal{N}}_{\text{in}}^{\dagger}(t')\Bigg]\}\rangle,
\end{aligned}
\tag{S35}
$$

which reads:

$$
\mathcal{P}_{\text{shot,shot}} = \kappa\eta\left(\tilde{n}+1/2\right)e^{-\kappa/2|t|}\left[e^{i\Delta t}+e^{-i\Delta t}\right], \tag{S36}
$$

where $\tilde{n} = (1-\eta)\,n_0 + \eta n_c$. This leads to the following noise floor:

$$
\mathcal{S}[\omega]_{\text{shot,shot}} = \eta\kappa^2\left(\tilde{n}+1/2\right)\left[\frac{1}{(\kappa/2)^2+(\omega-\Delta)^2}+\frac{1}{(\kappa/2)^2+(\omega+\Delta)^2}\right] \tag{S37}
$$

### 1.5.2 Mechanical noise

We have, in the limit of a high quality factor:

$$
\langle\hat{X}(t),\hat{X}(t')\rangle \approx \langle\hat{c}(t),\hat{c}^{\dagger}(t')\rangle + \langle\hat{c}^{\dagger}(t),\hat{c}(t')\rangle \tag{S38}
$$

This gives:

$$
\begin{aligned}
\langle\hat{X}(t),\hat{X}(t')\rangle \approx\ & \frac{\Gamma_m}{\Gamma_{\text{eff}}}e^{-\Gamma_{\text{eff}}/2|t'-t|}\left[\left(\bar{n}_{\text{th}}+1\right)e^{i\Omega_{\text{eff}}\left(t'-t\right)}+\bar{n}_{\text{th}}e^{-i\Omega_{\text{eff}}\left(t'-t\right)}\right] \\
& + e^{-\Gamma_{\text{eff}}/2|t-t'|}e^{-i\Omega_{\text{eff}}\left(t-t'\right)}\frac{g^2\kappa}{\Gamma_{\text{eff}}}\left\{|\mathcal{A}_+|^2\left(\tilde{n}+1\right)+|\mathcal{A}_-|^2\tilde{n}\right\} \\
& + e^{-\Gamma_{\text{eff}}/2|t-t'|}e^{i\Omega_{\text{eff}}\left(t-t'\right)}\frac{g^2\kappa}{\Gamma_{\text{eff}}}\left\{|\mathcal{A}_-|^2\left(\tilde{n}+1\right)+|\mathcal{A}_+|^2\tilde{n}\right\}
\end{aligned}
\tag{S39}
$$

This expression allows to compute the part of the spectrum associated with the mechanical noise. We have:

$$
\begin{aligned}
\mathcal{P}_{\text{mech,mech}}(t) = & \frac{g^2\kappa_c}{\Gamma_{\text{eff}}}e^{-\Gamma_{\text{eff}}|t|}\left(e^{i\Omega_{\text{eff}}t}+e^{-i\Omega_{\text{eff}}t}\right)\left(|\mathcal{A}_+|^2+|\mathcal{A}_-|^2\right) \\
& \times\left[\Gamma_m\left(n_m+1/2\right)+g^2\kappa\left(|\mathcal{A}_+|^2+|\mathcal{A}_-|^2\right)\left(\tilde{n}+1/2\right)\right]
\end{aligned}, \tag{S40}
$$

which in turn gives the following spectrum:

$$\mathcal{S}[\omega]_{\text{mech,mech}} = g^2\kappa_c \left( \frac{1}{(\Gamma_{\text{eff}}/2)^2 + (\omega-\Omega_{\text{eff}})^2} + \frac{1}{(\Gamma_{\text{eff}}/2)^2 + (\omega+\Omega_{\text{eff}})^2} \right) \left(|\mathcal{A}_+|^2 + |\mathcal{A}_-|^2\right) \\ \times \left[\Gamma_m\left(\bar{n}_{\text{th}}+1/2\right) + g^2\kappa\left(|\mathcal{A}_+|^2 + |\mathcal{A}_-|^2\right)\left(\tilde{n}+1/2\right)\right] \tag{S41}$$

### 1.5.3 Cross spectrum

The last part of the spectrum is the cross spectrum between the input optical noise and the backaction noise. We have:

$$\langle I(t), I(0)\rangle_{\text{cross}} = \langle I(t), I(0)\rangle_{\text{cross},1} + \langle I(t), I(0)\rangle_{\text{cross},2}, \tag{S42}$$

where:

$$\langle I(t), I(0)\rangle_{\text{cross},1} \underset{\text{def}}{=} \langle \sqrt{\kappa_c}\int_{-\infty}^{t} dt' e^{\kappa/2(t'-t)} \left[e^{-i\Delta(t'-t)}\hat{\mathcal{N}}_{\text{in}}(t') + e^{i\Delta(t'-t)}\hat{\mathcal{N}}_{\text{in}}^{\dagger}(t')\right], \\ g\sqrt{\kappa_c}\int_{-\infty}^{0} dt'' e^{\kappa/2t''}\left(-ie^{-i\Delta t''} + ie^{i\Delta t''}\right) \\ \Big\{ -ig\int_{-\infty}^{t''} dt''' e^{(\Gamma_{\text{eff}}/2+i\Omega_{\text{eff}})(t'''-t'')} \left(\mathcal{A}_+\hat{\mathcal{N}}_{\text{in}} + \mathcal{A}_-^*\hat{\mathcal{N}}_{\text{in}}^{\dagger}\right)(t''') \\ + ig\int_{-\infty}^{t''} dt''' e^{(\Gamma_{\text{eff}}/2-i\Omega_{\text{eff}})(t'''-t'')} \left(\mathcal{A}_+^*\hat{\mathcal{N}}_{\text{in}}^{\dagger} + \mathcal{A}_-\hat{\mathcal{N}}_{\text{in}}\right)(t''') \Big\}\rangle, \tag{S43}$$

and:

$$\langle I(t), I(0)\rangle_{\text{cross},2} \underset{\text{def}}{=} \langle g\sqrt{\kappa_c}\int_{-\infty}^{t} dt' e^{\kappa/2(t'-t)} \left(-ie^{-i\Delta(t'-t)} + ie^{i\Delta(t'-t)}\right) \\ \Big\{ -ig\int_{-\infty}^{t'} dt'' e^{(\Gamma_{\text{eff}}/2+i\Omega_{\text{eff}})(t''-t')} \left(\mathcal{A}_+\hat{\mathcal{N}}_{\text{in}} + \mathcal{A}_-^*\hat{\mathcal{N}}_{\text{in}}^{\dagger}\right)(t'') \\ + ig\int_{-\infty}^{t'} dt'' e^{(\Gamma_{\text{eff}}/2-i\Omega_{\text{eff}})(t''-t')} \left(\mathcal{A}_+^*\hat{\mathcal{N}}_{\text{in}}^{\dagger} + \mathcal{A}_-\hat{\mathcal{N}}_{\text{in}}\right)(t'') \Big\}, \\ \sqrt{\kappa_c}\int_{-\infty}^{0} dt''' e^{\kappa/2t'''}\left[e^{-i\Delta t'''}\hat{\mathcal{N}}_{\text{in}}(t''') + e^{i\Delta t'''}\hat{\mathcal{N}}_{\text{in}}^{\dagger}(t''')\right]\rangle, \tag{S44}$$

with similar definitions for $\langle I(0), I(t)\rangle$. The computation gives, in the resolved sideband and the weak coupling regime:

$$\mathcal{P}_{\text{mech,shot}}(t) = g^2\kappa^2\eta e^{-\Gamma_{\text{eff}}|t|/2}\left(e^{i\Omega_{\text{eff}}t} + e^{-i\Omega_{\text{eff}}t}\right)\left(\mathcal{A}_- - \mathcal{A}_+^*\right) \\ \times \left(|\mathcal{A}_-|^2 + |\mathcal{A}_+|^2\right)\left(\tilde{n}+1/2\right) \tag{S45}$$

which in turn gives the following spectrum:

$$\mathcal{S}[\omega]_{\text{mech,shot}} = g^2\kappa^2\eta \Big\{ \left( \frac{1}{(\Gamma_{\text{eff}}/2)^2 + (\omega-\Omega_{\text{eff}})^2} + \frac{1}{(\Gamma_{\text{eff}}/2)^2 + (\omega+\Omega_{\text{eff}})^2} \right) \\ \times \Gamma_{\text{eff}}\left(\mathcal{A}_- - \mathcal{A}_+^*\right)\left[\left(|\mathcal{A}_-|^2 + |\mathcal{A}_+|^2\right)\left(\tilde{n}+1/2\right)\right] \tag{S46}$$

## 1.6 Final result

In the case of a red detuned beam, in the resolved sideband regime, we have $\Delta \approx -\Omega_m$, $|\mathcal{A}_-|^2 \approx 0$, $|\mathcal{A}_+|^2 \approx 4/\kappa^2$ and therefore:

$$S[\omega] = n_{\mathrm{add}} + 4\eta\left(\tilde{n} + 1/2\right) + \eta\Gamma_{\mathrm{m}}\Gamma_{\mathrm{e}}\frac{\bar{n}_{\mathrm{th}} + \frac{1}{2} - \left(2 + \frac{\Gamma_{\mathrm{e}}}{\Gamma_{\mathrm{m}}}\right)\left(\tilde{n} + \frac{1}{2}\right)}{\left(\Gamma_{\mathrm{m}} + \Gamma_{\mathrm{e}}\right)^2/4 + \left(\omega - \omega_{\mathrm{p}} - \Omega_{\mathrm{eff}}\right)^2} \tag{S47}$$

.

# 2 System Summary

| | |
|---|---|
| Microwave cavity frequency | $\omega_c/2\pi = 8.350\,\mathrm{GHz}$ |
| Cavity total decay rate | $\kappa/2\pi = 226\,\mathrm{kHz}$ |
| Cavity out-coupling rate | $\kappa_e/2\pi = 183\,\mathrm{kHz}$ |
| Cavity internal loss rate | $\kappa_i/2\pi = 43\,\mathrm{kHz}$ |
| Cavity coupling efficiency | $\eta_c = 0.81$ |
| Mechanical frequency | $\Omega_m/2\pi = 1.487\,\mathrm{MHz}$ |
| Mechanical energy decay rate | $\Gamma_m/2\pi = 1.0\,\mathrm{mHz}$ |
| Mechanical quality factor | $Q_m = \Omega_m/\Gamma_m = 1.5\cdot 10^9$ |
| Electro-mechanical single photon-coupling rate | $g_0 = (0.89 \pm 0.11)\,\mathrm{Hz}$ |

Table S1: Experimentally measured electro-mechanical system parameters

The microwave device consists of a superconducting loop, whose dimensions are sketched in Fig. S1A. The loop features a gap on its bottom side which is bridged by a superconducting pad deposited on a membrane (see Fig.1A of the main text). The vertical distance of the pad to the loop electrodes is $d$: the exact value of $d$ will determine the microwave resonance frequency and the coupling of microwaves and mechanics. The device is placed in the vacuum volume of a metal box. The read-out of the microwave resonator is done by inductive coupling to a loop antenna (see Fig. S1B) which terminates a coaxial cable (grounded at the box) leading to our instrumentation.

## 2.1 Finite element simulations

In the commercial finite element solver COMSOL, we simulate the microwave device to estimate the resonance frequency. The geometry is depicted Fig. S2A: the loop-gap resonator is on a silicon substrate (highlighted in light blue). The fundamental mode of the loop (depicted in Fig. S2B) has currents flowing back and forth around the loop with the largest electric field across the pad/electrode gap. The concentration of electric field at the location of the mechanical element allows for high electro-mechanical coupling.

In the simulations, we vary the membrane-pad-to-electrodes distance $d$ and fit its effect on the resonance frequency (Fig. S2C). As $d$ is reduced, the parallel-plate capacitance $C_{\mathrm{m}}$ formed by the pad and the electrodes increases which pulls down the microwave frequency $\omega_{\mathrm{r}}$. We model the mechanical capacitance to be in parallel with

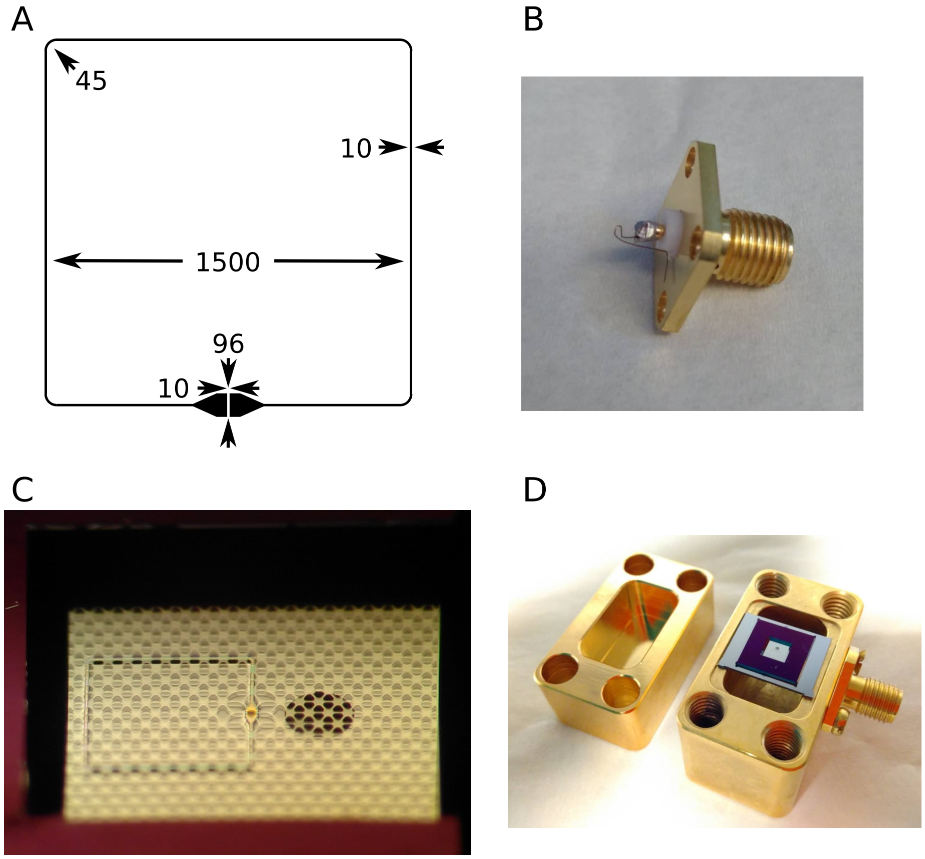


Figure S1: A) Loop-gap resonator mask with dimension in microns. B) SMA connector for inductive in-coupling to the microwave cavity: one end of the hand-wound loop is soldered to the central pin of the connector, while the other end is clamped on the sample holder for grounding. C) Zoom-in at an angle of the assembled flipchip with the patterned membrane hovering over the loop-gap resonator. D) The flipchip is placed in the middle of a hollow volume of a copper (gold-coated) sample holder. The inductive in-coupling loop (shown in B) protrudes into the hollow volume underneath the resonator to achieve the mutual inductive coupling.

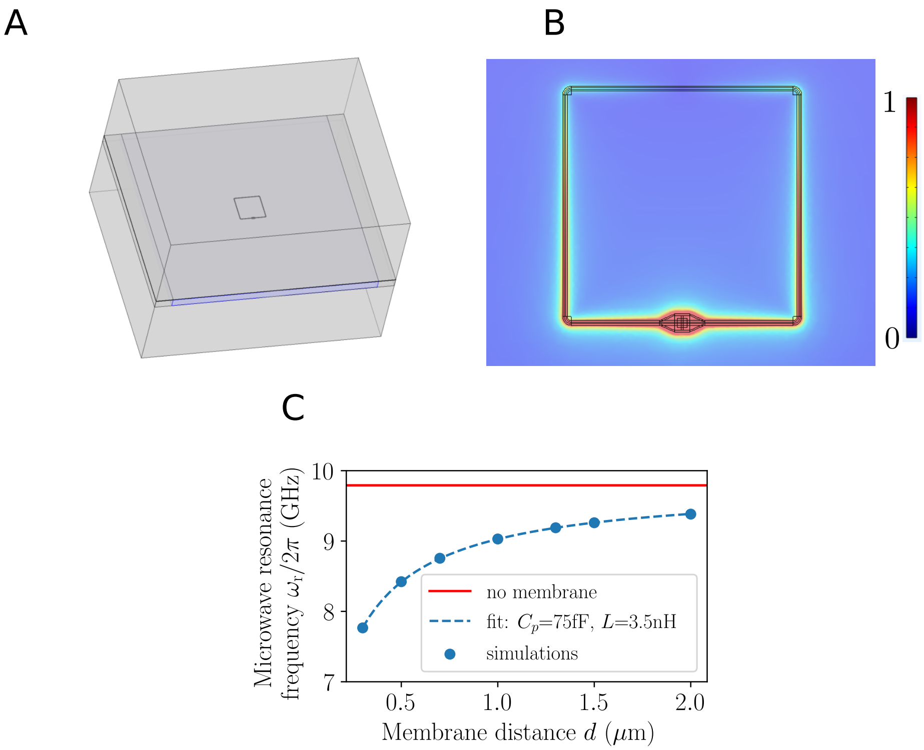


Figure S2: A) Finite element geometry to simulate the microwave mode. B) Simulated electric field norm for the loop fundamental mode. C) As the membrane distance is reduced, the microwave resonance frequency is pulled-down. Simulated frequencies are fitted with Eq. (S48).

the loop's own capacitance $C_\mathrm{p}$, this total capacitance with the loop inductance forms the LC resonator with frequency

$$\omega_\mathrm{r} = \frac{1}{\sqrt{[C_\mathrm{m}(d) + C_\mathrm{p}]L}}. \tag{S48}$$

The fit of simulated frequencies allows us to extract the parasitic capacitance $C_\mathrm{p}$: for the fabricated geometry, we simulate $C_\mathrm{p} \approx 75\,\mathrm{fF}$. In absence of membrane pad, the simulated loop resonance is at approx. 9.8 GHz.

From the measured microwave frequency in Fig. S3 $\omega_\mathrm{r} = 8.349\,\mathrm{GHz}$, we can estimate $d \approx 450\,\mathrm{nm}$. The measured participation ratio of capacitances $C_\mathrm{m}/(C_\mathrm{m} + C_\mathrm{p}) \approx 0.25$.

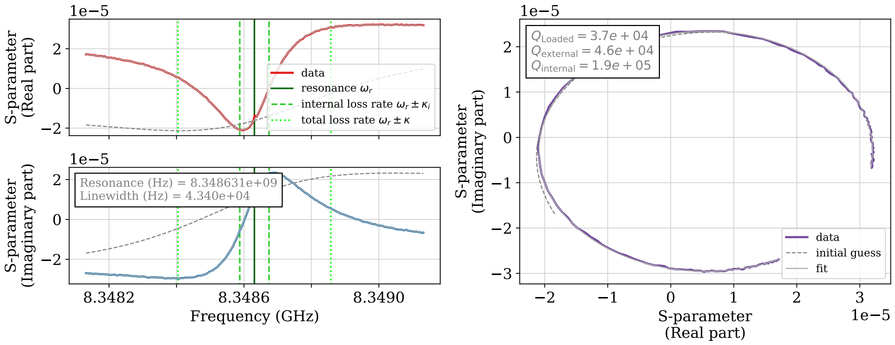


Figure S3: Example of a cavity fit: here for the data point at 0 dBm from Fig.3D in the main text, where the mechanics is prepared in its ground state.

# 3 Calibrations

## 3.1 Example of a cavity fit

In Fig. S3, we plot the measured cavity reflection coefficient as function of frequency and fit it to extract cavity parameters. This spectroscopy allows gives us the parameters in Table S1, and is taken for the data point in Fig. 3D, where the mechanics is prepared in its ground state.

## 3.2 Noise photons in the cavity

To confirm the origin of the increased background level around the mechanical feature (as seen in Fig. 3B of the main text), which leads to noise squashing, we plot the wider power spectrum around the mechanical bandgap in Fig. S4 corresponding to the ground state preparation data point in Fig. 3D of the main text. A wide feature appears at the cavity frequency which we overlay with a cavity lineshape function $\mathrm{ls}(\omega) \propto [1+(\omega-\omega_\mathrm{r})^2/\kappa^2]^{-1}$ on top of the instrument background. In this sideband spectrum, the cavity appears in the mechanical bandgap at the location of the mechanical mode frequency because the pump is red-detuned by $\Omega_\mathrm{m}$. In this lineshape function we use $\omega_\mathrm{r}$ and $\kappa$ from the vector network analyser fit in Fig. S3. As reference, we also plot a low pump power spectrum from which we extract the instrument noise background.

Following ref. [2], we can interpret the excess noise as a relative phase noise between the pump and the cavity, and obtain a value of this phase noise to be approx. -145 dBc/Hz, which is about 10 dB higher than the phase noise as specified (at 1 MHz sideband frequency) by the manufacturer of our low noise signal generator. This discrepancy makes us suspect that the dominant phase noise actually stems from the cavity, such as cavity frequency jitter. Indeed we have tried adding a microwave notch filter at the exit of the signal generator, reducing its phase noise by an additional 8 dB, but this did not lead to improved cooling limit of the mechanics.

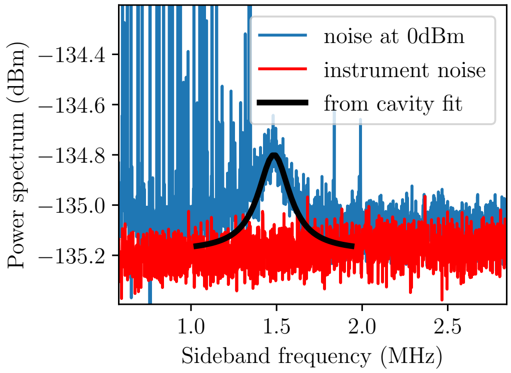


Figure S4: Spectrum at the mechanical bandgap at high pump power. At high powers, a wide feature appears which has the lineshape of the cavity. This cavity feature results from cavity population, which could be caused by relative frequency fluctuations of the pump and the cavity.

## 3.3 Additional mechanical dephasing

The high signal-to-noise of the ringdown measurements presented in Fig.2 of the main text allows us to also look at the frequency stability of the mechanical resonator. We specifically look at the phase $\phi(t)$ of the three ringdowns at powers $-60$, $-50$ and $-40$ dBm and compute their instantaneous frequency $d\phi(t)/dt$ (with respect to the spectrum analyser's local oscillator) over the $\approx$600 s of the ringdown. We choose these three ringdowns since they are the only ones of this measurement series which are far above the noise background for the entirety of the ringdown.

In Fig.S6A, we plot the instantaneous mechanical frequency over time (averaged over 10 s for each data point) of each ringdown and fit them by an affine function $f(t) = d_0 t + f_0$, with $d_0$ the drift over time and $f_0$ the initial frequency. We extract a small drift of 2.7 µHz s$^{-1}$ on all three ringdowns, corresponding to a drift 1.6 mHz after 600 s. The extracted $f_0$ agree within 3 mHz, consistent with the drift.

We have not taken spectra with enough frequency resolution to plot the mechanical spectrum and extract spectral linewidth for the device in this paper. However on a different device, we have compared a large signal-to-noise ratio mechanical spectrum with an energy ringdown measurement in the same conditions. Spectrum and ringdown for this second device are plotted in Fig.S6B and C respectively. The linewidth from the Lorentzian spectral fit and the energy decay rate from ringdown are $(2.60 \pm 0.15)$ mHz and $(2.13 \pm 0.02)$ mHz: thus any additional dephasing makes up at most 22% of the total mechanical linewidth. This second device in question had a mechanical frequency of $\Omega_\mathrm{m}/2\pi = 1.487$ MHz and therefore a spectral and ringdown quality factor of $570 \pm 33$M and $696 \pm 5$M.

## 3.4 Gorodetsky calibration

To calibrate the single photon coupling rate $g_0$, we use the so-called "Gorodetsky method" [3] where we compare the phase-modulation imparted by the mechanical mod-

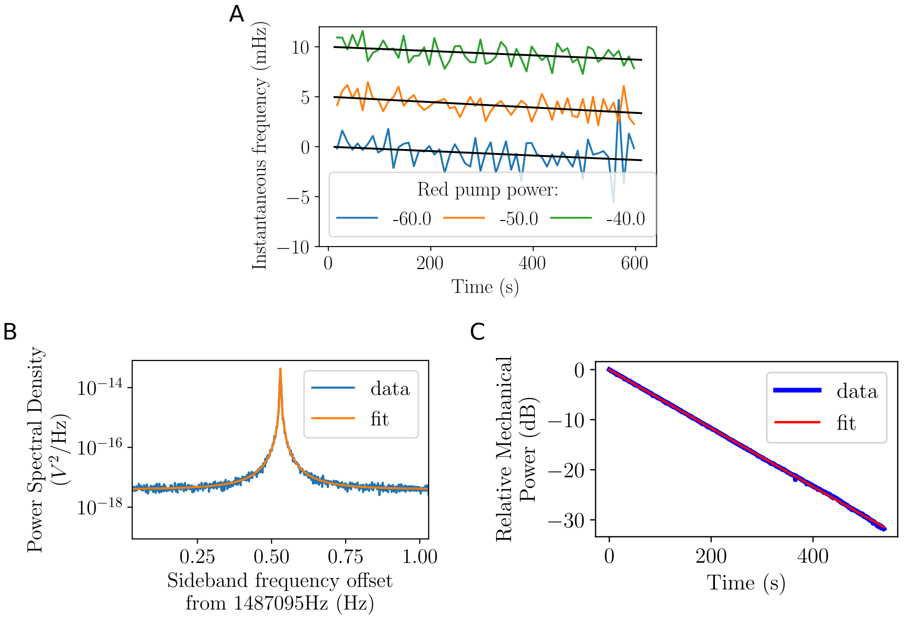


Figure S5: A) Instantaneous frequency during ringown plotted over time as offset from its value $f_0$ at time at $t = 0$, traces are offset by 5 mHz for clarity. A small drift of the mechanical frequency is visible, the drift after 600 s is at most 1.6 mHz. B) Mechanical spectrum of a second device fitted with a Lorentzian function gives a linewidth of $(2.60 \pm 0.15)$ mHz. C) Energy ringdown for the second device taken in the same conditions as the spectrum yields a decay rate of $(2.13 \pm 0.02)$ mHz.

ulation of the cavity frequency to the pump with a known reference phase-modulation (See Fig. S6A). This method requires that the amount of mechanical quanta is known, which we extract by confirming that the mechanics is in a thermal state. The number of quanta in the mechanical thermal state can then be computed using the Bose-Einstein relation.

The Gorodetsky calibration is set up as an unbalanced homodyne measurement. We phase modulate the pump internally in the signal generator, split it with a 3dB splitter where half of the signal goes to the electro-mechanics cavity and the other half is used as the homodyne local oscillator. Attenuation in the dilution refrigerator is large such that the LO amplitude is much higher than the signal returned from the microwave cavity. We additionally use a variable phase shifter on the LO to optimise the interference of cavity signal with LO. A wiring diagram is shown in Fig. S10A. The homodyne measurement is saved as the I and Q quadratures of the electric field, for which we numerically take the modulus squared to plot the power spectrum, as in Fig. S6A.

We apply a pump on cavity resonance, red-detuned by a few kHz, such that, if there is any dynamical backaction, the mechanics does not become unstable and instead is slightly broadened. We monitor the amount of backaction by measuring a ringdown at the pump power of the spectral measurement and compare it to a ringdown at low power, where we know the mechanics to not be broadened (See Fig.S6B).

In Fig.S6C we plot the ratio of mechanical peak area to calibration peak area, extracted from spectra such as in Fig.S6A. At each temperature point this ratio is multiplied by $(\Gamma_{\mathrm{opt}} + \Gamma_{\mathrm{m}})/\Gamma_{\mathrm{m}}$ corresponding to the cooling factor due to dynamical backaction. The linear relationship of measurement points to thermometer reading $T$ at high temperatures indicates the mechanics is thermalised at least above 200 mK. We can use this thermalisation condition of extract the number of mechanical quanta with $\bar{n} \approx k_{\mathrm{B}}T/\hbar\Omega_{\mathrm{m}}$, with $k_{\mathrm{B}}$ Boltzmann's constant and $\hbar$ Planck's constant divided by $2\pi$. Below 200 mK, the mechanical area has a large scatter which the authors in Ref. [4] attribute to a yet unknown force which appear at very low temperatures.

The calibration yields $g_0 = (0.89 \pm 0.11)\,\mathrm{Hz}$.

### 3.5 Cavity monitoring

In the measurement series for thermal calibration in Fig. 2A of the main text as well as for the ground state cooling in Fig. 3, we monitor the cavity parameters for each temperature and power point. Cavity parameters are plotted as function of temperature in Fig. S7 and as function of power at base temperature in Fig. S8. We then use these updated parameters to extract the mechanical occupation at each temperature and power.

At higher pump powers in Fig. S8, we see a reduction of the internal loss and we see a logarithmic dependence of the resonance frequency to power. Both of effect suggest the presence of two-level systems in the surrounding material coupling to the microwave field[5]. Furthermore these measurements confirm the cavity remains linear at all pump powers: that is the kinetic inductance of the NbTiN thin film is too small to impact the resonance frequency. If there were a kinetic inductance non-linearity, the resonance frequency would go linearly with power and not logarithmically.

Additionally, we do not see a significant increase in internal cavity loss as the sample temperature moves towards the superconducting transition temperature (around 1 K) of the Al membrane metallisation. A low participation ratio of mechanically-mediated capacitance to total capacitance could explain why the microwave losses are

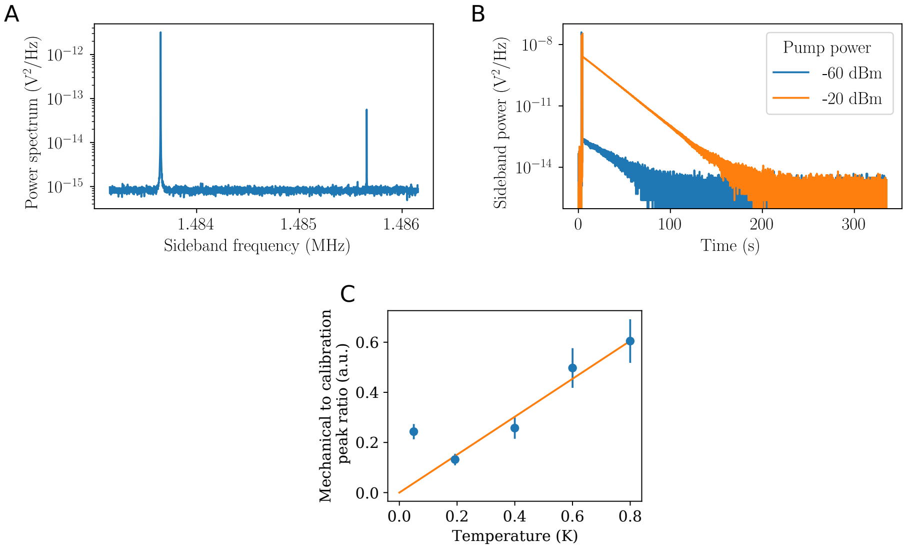


Figure S6: A) An example spectrum with phase-modulated calibration peak (lower frequency) and mechanical thermal peak (upper frequency) at 800 mK and -20 dB drive B) Mechanical ringdown measurements without dynamical backaction (blue) and at the pump power of the spectrum in A (orange), where there is a small amount of backaction. C) Ratio of mechanical peak area to calibration peak area for varying sample temperature, where we adjust for any dynamical backaction using the ringdown measurements, such as in B. The line is a linear fit to the higher temperature points, confirming thermalisation of the mechanics to the environment above 200 mK. Error bars correspond to one std. dev. of the Lorentzian fit.

not affected by the superconducting state of the aluminum.

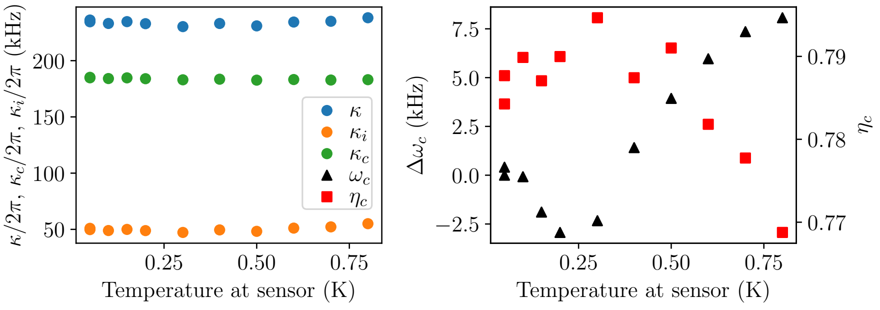


Figure S7: Changes in cavity parameters as the sample temperature is varied, during the calibration sequence. Cavity scans are taken with −45 dBm output from the signal generator.

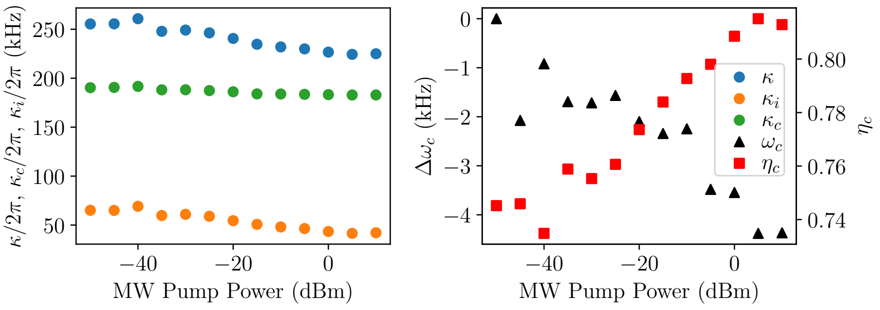


Figure S8: Changes in cavity parameters as the pump power is increased, during the ground state cooling series. All cavity measurements are taken at 30 mK.

## 3.6 Mechanical mode damping

We measure the mechanics' intrinsic decay rate as function of thermometer temperature in Fig. S9. We fit the data to a power law of temperature $\propto (T/T_0)^\alpha$, with $T_0$ and arbitrary reference temperature and the exponent $\alpha$. For the two analysed mechanical modes, the power law relations with $\alpha = 0.63$ and $\alpha = 0.76$ are consistent with mechanical two level systems (TLS) coupling to the mode of interest and extracting mechanical energy from it[4].

We point out that since the mechanical mode in Fig. S9B at 2.671 MHz is located in the second mechanical bandgap, its spatial extend is less that for the 1.486 MHz mode, located in the first mechanical bandgap. Therefore the Al metallisation makes up a larger fraction of its out-of-plane displacement profile. The larger exponent $\alpha = 0.76$ could thus be attributed to the larger relative amount of Al in the mechanical mode.

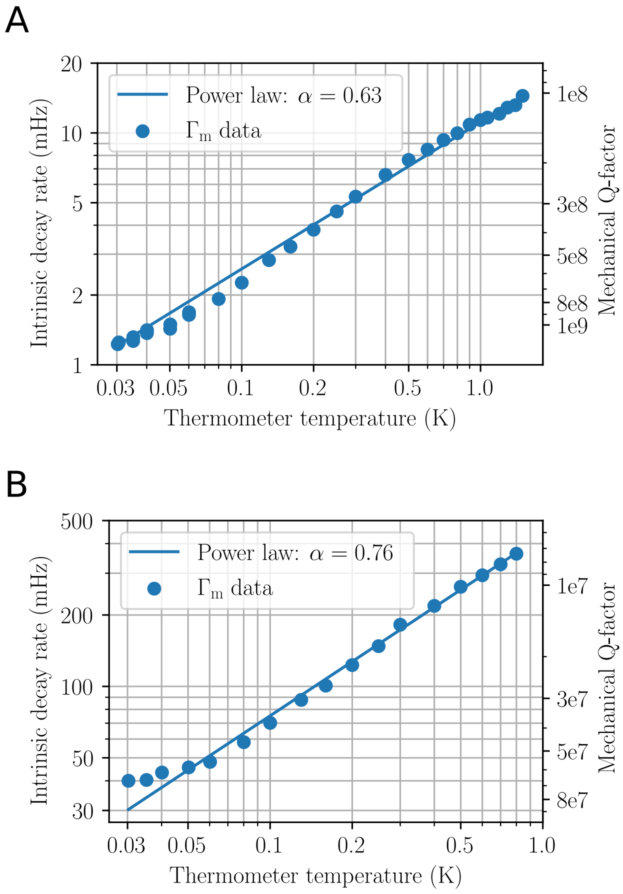


Figure S9: Intrinsic mechanical decay rate as function of thermometer temperature. The power law fits indicate that coupling to mechanical TLS is the dominant loss mechanism for mechanical energy. We plot losses for two mechanical modes of the device: A) is the mode at 1.486 MHz discussed in the rest of the manuscript; B) is a mode at 2.671 MHz, located in a higher mechanical bandgap.

## 3.7 Cryogenic setup

In Fig. S10 A, we show a diagram of the microwave cable wiring used in this experiment. Fig. S10B is a picture of the mechanical damper to which the electromechanical assembly is mounted. The damper consists of a oxygen-free high-conductivity copper block suspended from the mixing chamber plate by steele springs. This mass-on-a-spring setup acts as a mechanical low pass filter damping any environmental vibrations at the mechanical frequency. Each of the three springs has a spring constant of 0.1 N/mm and the copper block has a mass of 1.4 kg, thus with the three springs in parallel we expect a low-pass filter corner frequency of ≈0.5 Hz, above which the

suppression of vibration goes as $f^{-2}$.

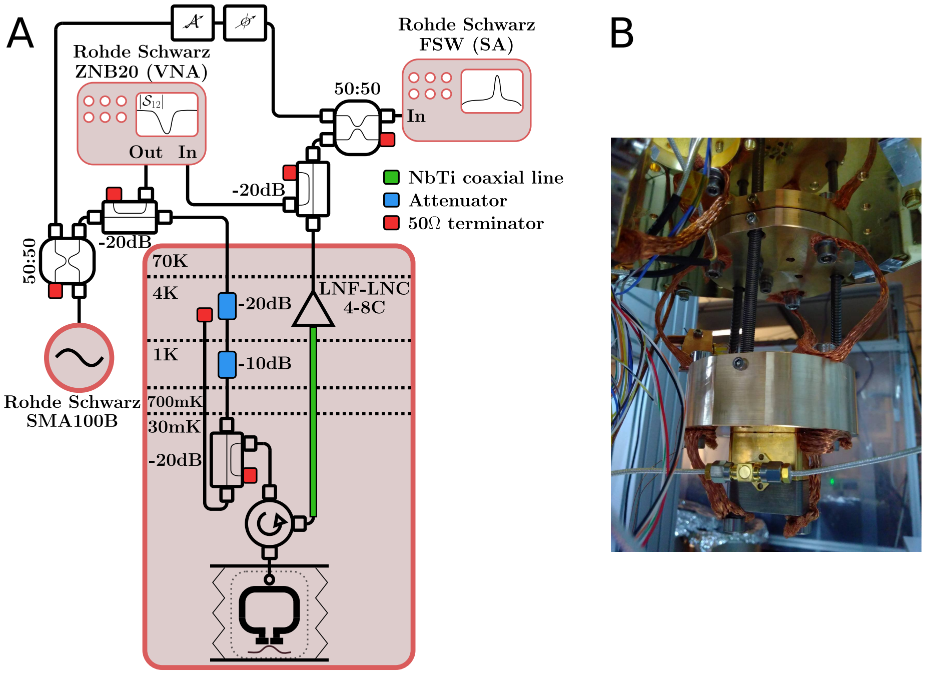


Figure S10: A) Setup of the microwave equipment around the dilution refrigerator. B) Photograph of the single-stage mechanical damper mounted to the mixing chamber plate (top) of the dilution refrigerator to isolate from environmental vibrations, especially the pulse tube.

## 3.8 Calibration consistency check

In order to verify that the thermal calibration, on which relies the core result of the manuscript, is correct, we performed two separate consistency checks:

- Given the company specified gain and noise temperature of the HEMT at the operating frequency, we calibrated the spectrum data at low power from figure 4 of the main text, using the background noise as a reference. This translates, given the independent knowledge we have of the overcoupling ratio, the cooperativity and the mechanical linewidth, into a measured mechanical occupation, which is associated with a lower temperature than the extracted 80mK temperature bath. The ratio between those two temperatures is due to the attenuation between the sample and the HEMT amplifier. We found this attenuation to be approximately 6.0 dB. Separately, we summed the company specified losses of all the components, cables and connectors between the sample and the HEMT, and obtained 6.2 dB, with an estimated systematic error of ±1.5 dB, given the limited applicability of the specifications to our low-temperature setting combined with the impossibility to accurately measure them at low temperature, the

large number of components present and the uncertainties in connector losses for instance.

- Alternatively, the thermal calibration provides a measurement of the gain between the sample and the analyzer, and combined with a transmission measurement of the whole setup, it allows to compute the attenuation between the source and the sample. We found this attenuation to be 66.5 dB, and separately, summing again the company specified losses of all the components, we found 65.5 dB of attenuation with an estimated systematic error of $\pm 4$ dB.

Those two consistency checks strengthen our confidence in the thermal calibration presented in Fig 3 of the main text.

# References


[1] Weinstein, A. J. *et al.* Observation and interpretation of motional sideband asymmetry in a quantum electromechanical device. *PRX* **4**, 041003 (2014).

[2] Yuan, M., Singh, V., Blanter, Y. M. & Steele, G. A. Large cooperativity and microkelvin cooling with a three-dimensional optomechanical cavity. *Nature Communications* **6**, 8491 (2015).

[3] Gorodetksy, M. L., Schliesser, A., Anetsberger, G., Deleglise, S. & Kippenberg, T. J. Determination of the vacuum optomechanical coupling rate using frequency noise calibration. *Opt. Express* **18**, 23236–23246 (2010).

[4] Zhou, X. *et al.* On-chip thermometry for microwave optomechanics implemented in a nuclear demagnetization cryostat. *Phys. Rev. Applied* **12**, 044066 (2019).

[5] Capelle, T. *et al.* Probing a two-level system bath via the frequency shift of an off-resonantly driven cavity. *Phys. Rev. Applied* **13**, 034022 (2020).
